# Supplementary material for: Application of ATR-FT-MIR for Tracing the Geographical Origin of Honey Produced in the Maltese Islands
Source: Foods. 2020 Jun 1;9(6):710. doi: 10.3390/foods9060710 (PMC7353483; doi:10.3390/foods9060710)
Supplement: Supplementary file 1 [file foods-09-00710-s001.docx]

## S.1 Honey samples

### Local samples

Samples which are described as local in this study were collected directly from Maltese and Gozitan beekeepers post honey harvest during 2015 and 2016. A detailed table with the locality and botanical origin can be found below (Table S.1). The localities of collection are mapped out in Figure S.1.

Table 1. Sample code, locality and date of harvest for local samples.

| **ID** | **Locality** | **Botanical Origin** |
| --- | --- | --- |
| **M1** | Malta, Birzebbugia | Carob and Eucalyptus |
| **M2** | Malta, Mellieha | Multifloral |
| **M3** | Malta, Siggiewi | Carob and Eucalyptus |
| **M4** | Malta, Wardija | Multifloral |
| **M5** | Malta, Gargur | Carob and Eucalyptus |
| **M6** | Malta, Siggiewi | Multifloral |
| **M7** | Malta, Fawwara | Thyme |
| **M8** | Malta, Fawwara | Multifloral |
| **M9** | Malta, Wardija | Thyme |
| **M10** | Malta, Wardija | Carob and Eucalyptus |
| **M11** | Malta, Gargur | Multifloral |
| **M12** | Malta, Mgarr | Multifloral |
| **M13** | Malta, Zebbiegh | Multifloral |
| **G1** | Gozo, Nadur | Multifloral |
| **G2** | Gozo, Ghasri | Thyme |
| **G3** | Gozo, Ghasri | Multifloral |
| **G4** | Gozo, Nadur | Thyme |
| **G5** | Gozo, Marsalforn | Multifloral |
| **G6** | Gozo, Ghajsielem | Multifloral |
| **G7** | Gozo, Nadur | Multifloral |
| **G8** | Gozo, Xaghra | Thyme |


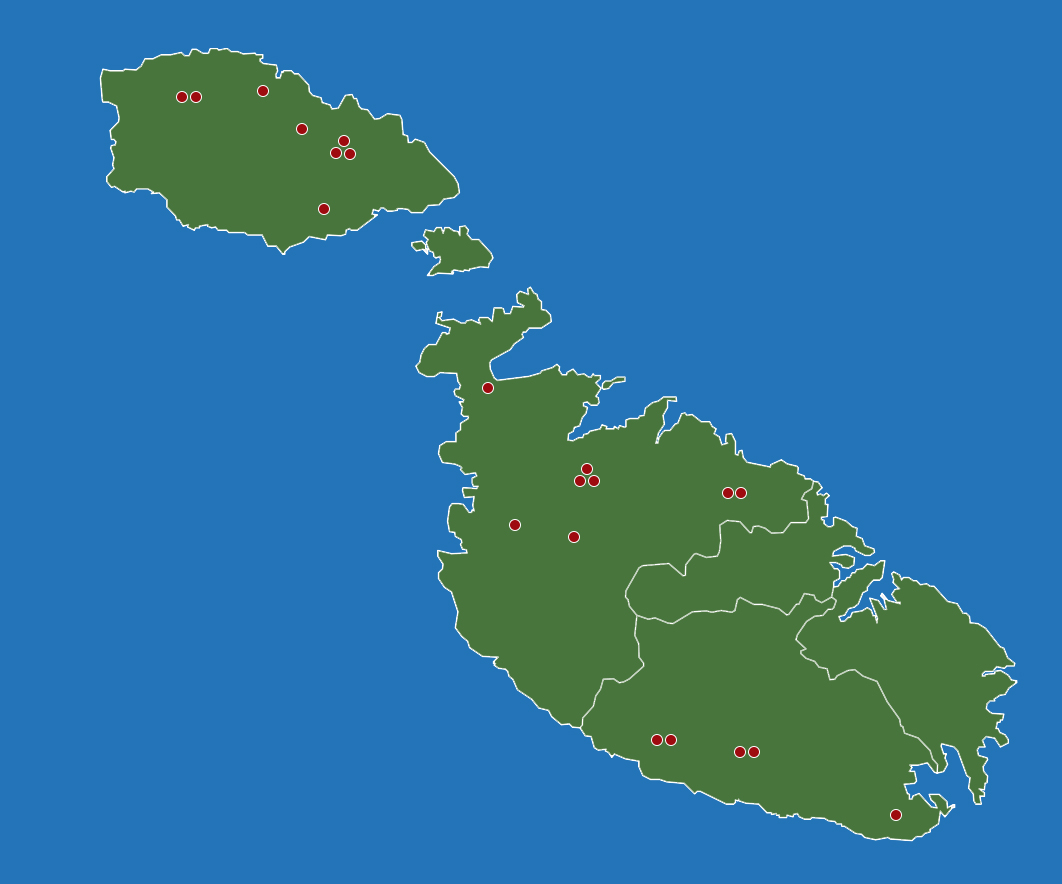


Figure 1. Map of Maltese islands highlight locality of honey samples in Table 2.1.

## S.2 PCA

PCA identified no outliers within the dataset for the untreated spectra or when the spectra were subjected to spectral transformations. Through PCA, no samples were observed to cluster according to geographical origin (Figure 2). This result is not unexpected since the honey samples being tested do not differ only by geographical origin, since other sources of variability such as botanical origin were present. The loading plot in Figure 3 also suggests that the data is modelling spectral features rather than noise present in the spectra.

Figure 2. Two component plot from PCA on Median filtered data in the region between 760 and 1400 cm^-1.^

Figure 3: Loading plot for the first two component of PCA on Median filtered data in the region between 760 and 1400 cm^-1^

Marion Zammit-Mangion, Marina Meixner, David Mifsud, Sheryl Sammut & Liberato Camilleri (2017) Thorough morphological and genetic evidence confirm the existence of the endemic honey bee of the Maltese Islands *Apis mellifera ruttneri*: recommendations for conservation, Journal of Apicultural Research, 56:5, 514-522, DOI: [10.1080/00218839.2017.1371522](https://doi.org/10.1080/00218839.2017.1371522)

1. Attard, E. Bugeja Douglas, A. Physicochemical Characterization of Maltese Honey, Honey Analysis, Prof. Vagner Arnaut De Toledo (Ed.), InTech, 2017
2. [Apis mellifera ruttneri, a new honey bee subspecies from Malta](https://www.apidologie.org/articles/apido/abs/1997/04/Apidologie_0044-8435_1997_28_5_ART0005/Apidologie_0044-8435_1997_28_5_ART0005.html) W.S. Sheppard, M.C. Arias, A. Grech, M.D. Meixner Apidologie, 28 5 (1997) 287-293
3. Everaldo Attard* and Joseph Mizzi Physicochemical characterization of Gozitan Honey International Journal of Food Studies IJFS October 2013 Volume 2 pages 180–187
4. Attard, E., Bugeja Douglas, A., & Camilleri, Ch. (2013). Molecular characterization of Maltese honey : diastase and proline levels changes in Maltese honey seasons. Farm Animal Proteomics 2013, Košice. 266-269.
5. Attard, E., & Bugeja Douglas, A. (2017). Physicochemical characterization of Maltese honey. In Honey Analysis. Croatia: InTech.
6. Reema Valand, Sangeeta Tanna, Graham Lawson & Linda Bengtström (2020) A review of Fourier Transform Infrared (FTIR) spectroscopy used in food adulteration and authenticity investigations, Food Additives & Contaminants: Part A, 37:1, 19-38,
